# Efficient and Privacy-Preserving Power Distribution Analytics Based on IoT

**DOI:** 10.3390/s25216677

**Published:** 2025-11-01

**Authors:** Ruichen Xu, Jiayi Xu, Xuhao Ren, Haotian Deng

**Affiliations:** School of Cyberspace Science and Technology, Beijing Institute of Technology, Beijing 100081, China13013208068@163.com (X.R.)

**Keywords:** Internet of Things, power distribution analytics, Hilbert curve, distributed point functions

## Abstract

The increasing global demand for electricity has heightened the need for stable and reliable power distribution systems. Disruptions in power distribution can cause substantial economic losses and societal impact, underscoring the importance of accurate, timely, and scalable monitoring. The integration of Internet of Things (IoT) technologies into smart grids offers promising capabilities for real-time data collection and intelligent control. However, the application of IoT has created new challenges such as high communication overhead and insufficient user privacy protection due to the continuous exchange of sensitive data. In this paper, we propose a method for power distribution analytics in smart grids based on IoT called PSDA. PSDA collects real-time power usage data from IoT sensor nodes distributed across different grid regions. The collected data is spatially organized using Hilbert curves to preserve locality and enable efficient encoding for subsequent processing. Meanwhile, we adopt a dual-server architecture and distributed point functions (DPF) to ensure efficient data transmission and privacy protection for power usage data. Experimental results indicate that the proposed approach is capable of accurately analyzing power distribution, thereby facilitating prompt responses within smart grid management systems. Compared with traditional methods, our scheme offers significant advantages in privacy protection and real-time processing, providing an innovative IoT-integrated solution for the secure and efficient operation of smart grids.

## 1. Introduction

The rapid advancement of smart grid technology has profoundly reshaped global electricity distribution and consumption patterns [1,2,3]. The incorporation of renewable energy resources, distributed generation units, and sophisticated communication infrastructures has markedly enhanced the efficiency and reliability of modern power systems. Nevertheless, this progression also poses new challenges in monitoring and analyzing power distribution across expansive and complex grids. With the diversification of energy sources, the increasing dynamism of consumption behaviors, and the growing structural complexity of power networks, ensuring efficient power distribution analysis and maintaining grid stability have become pivotal responsibilities for electricity management authorities and grid operators.

The reliable and optimal operation of smart grids fundamentally depends on efficient monitoring and thorough analysis of power distribution [4,5]. A robust monitoring system is the key to accurately assessing the distribution of electricity across different regions. Meanwhile, power distribution analysis, which involves identifying the overall distribution patterns of electricity across the grid, constitutes a key component of smart grid monitoring. Understanding how power is distributed allows grid operators to optimize energy flow, improve grid efficiency, and identify areas with potential distribution issues. Without efficient monitoring and effective distribution analysis, inefficiencies and disruptions such as load imbalances, voltage fluctuations, or localized energy shortages can result in increased operational costs, energy wastage, and consumer dissatisfaction, leading to significant economic losses and affecting critical services [6]. Traditional power distribution methods, such as load flow analysis [7,8] or voltage regulation techniques [9], attempt to provide detailed distribution information. While useful in certain scenarios, these methods can face challenges when applied to large, complex, or dynamically changing grids. Therefore, enhancing power distribution analysis systems to ensure accurate, real-time insights is extremely important for maintaining the efficiency and stability of modern power grids [10].

The Internet of Things (IoT) offers a promising approach to addressing the challenges of efficient monitoring and analysis of power distribution in smart grids [11,12,13,14]. By connecting a vast network of sensors, devices, and control units, IoT facilitates real-time data collection and system-wide visibility, enabling systems to make informed decisions and adapt to dynamic changes in energy usage. However, the integration of IoT into smart grids still requires effective resource management strategies to maintain operational stability. Furthermore, the significant increase in data exchange introduces new challenges related to communication overhead and privacy protection, emphasizing the need for secure and scalable IoT architectures tailored for smart grids [15,16,17,18].

To address these challenges, this paper introduces PSDA (Power Distribution Analytics Based on IoT), a novel scheme that combines IoT, advanced encoding techniques, and privacy-preserving technologies for power distribution analysis. Our solution leverages an IoT system to monitor power distribution across the grid, applying Hilbert curve-based encoding to efficiently partition regions and reduce communication overhead. Additionally, we incorporate distributed point functions (DPFs) to preserve privacy and ensure security of the transmitted data. Our contributions are as follows:

- We propose the PSDA scheme to address the challenges of power distribution analysis in smart grids. Our control solution is based on an IoT system, supporting efficient monitoring and analysis of power distribution.
- We apply Hilbert curve-based encoding to efficiently manage IoT devices, optimizing the processing and representation of spatial regions. Furthermore, we incorporate DPF techniques to guarantee the privacy and security of the power distribution data. This combination of advanced encoding and privacy-preserving techniques enhances the accuracy and security of the distribution analysis while addressing privacy concerns in smart grid applications.
- The experimental results substantiate the superior effectiveness of the proposed method, demonstrating consistent performance improvements over existing approaches. The results indicate that PSDA achieves superior performance in both computational efficiency and communication overhead, offering a robust solution for analyzing power distribution in contemporary power systems.

The remainder of this paper is structured as follows. We systematically review and categorize the prevailing approaches to privacy preservation in smart grids in Section 2. Section 3 provides the necessary background knowledge, while Section 4 introduces the system models and design objectives. The proposed scheme is described in detail in Section 5. Section 6 presents the experimental results, followed by a review of related work in Section 7. Finally, Section 8 concludes the paper.

## 2. Related Work

With the rapid evolution of smart grid infrastructures, ensuring data privacy and system efficiency has become a primary research challenge. The integration of Internet of Things (IoT) devices has enabled fine-grained monitoring and real-time control of power distribution networks, yet it also introduces potential risks to data confidentiality and communication overhead. To address these challenges, numerous privacy-preserving approaches have been proposed over the past few years, focusing on secure data aggregation, authenticated communication, and privacy-aware analytics in IoT-based smart grids.

Early works primarily relied on lightweight encryption and access control to protect measurement data collected from smart meters and sensors. These approaches achieved basic confidentiality and integrity but often lacked scalability when applied to large-scale, heterogeneous IoT environments. Subsequently, differential privacy (DP) was introduced as a means to perturb energy consumption data before aggregation, effectively concealing individual usage patterns from adversaries [2,16]. Although DP-based methods [17] can guarantee formal privacy bounds, the introduction of statistical noise inevitably degrades the accuracy of aggregated results, which limits their applicability in scenarios that demand precise power distribution analytics or real-time control decisions. Beyond classical perturbation-based defenses, recent studies show that adding random noise to intermediate features may not robustly prevent information leakage against sophisticated model-stealing adversaries; for example, BESA demonstrates perturbation recovery that undermines noise-based defenses [19], underscoring the need for stronger cryptographic or protocol-level safeguards in smart grid analytics.

In response to the inherent accuracy–privacy trade-off of DP, researchers have increasingly explored exact encryption methods capable of preserving data precision while ensuring confidentiality during computation and transmission. For instance, Rani et al. [20] developed an encryption-based fog-assisted smart grid system that reduces communication latency by processing encrypted data near the edge, improving privacy and responsiveness simultaneously. Rostampour et al. [21] proposed a privacy-enhanced authentication protocol for IoT-based smart grid infrastructures, ensuring end-to-end confidentiality and integrity of transmitted measurements. These encryption-centric frameworks maintain accurate results by avoiding perturbation noise, aligning with the operational demands of modern grid analytics. However, a significant challenge for such encryption-centric frameworks lies in the limited computational resources of distributed IoT devices (e.g., smart meters), which often struggle to perform complex cryptographic operations efficiently. At the protocol layer, privacy-preserving and efficient asynchronous payment designs on blockchain, such as Epass [22], illustrate how to minimize online interaction and on-chain overhead while protecting transaction unlinkability—design principles that can inform secure, low-latency metering, settlement, and P2P energy trading modules within smart grids.

Furthermore, the emergence of privacy-preserving data aggregation frameworks has accelerated research on distributed computation architectures. Recent studies have adopted federated or multi-party learning paradigms to analyze grid data without directly sharing raw measurements among entities [5,15]. These systems enable local computation and global aggregation while reducing central communication burdens. However, they often require complex synchronization and model-sharing protocols, potentially incurring additional system latency. Complementary insights arise from mobile networking scenarios, that privacy-preserving federated learning frameworks tailored to cloud systems emphasize personalized aggregation, secure aggregation, and resource-aware client selection to bolster robustness and efficiency [23]. These techniques can be adapted to smart-grid Federated Learning deployments where data distributions vary across feeders, neighborhoods, and device types.

In contrast to existing privacy-preserving smart grid frameworks, the proposed study introduces an innovative combination of distributed point functions (DPFs) and Hilbert curve encoding, offering a lightweight yet secure and accurate approach to privacy protection. Unlike traditional encryption methods or differential privacy (DP) techniques, which either impose significant computational overhead or introduce inaccuracies due to noise injection, the integration of DPF with Hilbert curve encoding ensures both efficient computation and precise data aggregation. The clever use of Hilbert curves not only enhances the efficiency of DPF’s aggregation process but also links the encoded data to the geographical locations of IoT devices. This spatially aware encoding provides a scalable solution for real-time, privacy-preserving analytics in IoT-enabled smart grids, addressing the operational needs of next-generation grid systems with minimal communication costs and full data accuracy.

## 3. Materials

In this section, we introduce some background preliminaries related to our scheme, including Hilbert curves and distributed point functions.

### 3.1. Hilbert Curve

**Figure 1:**
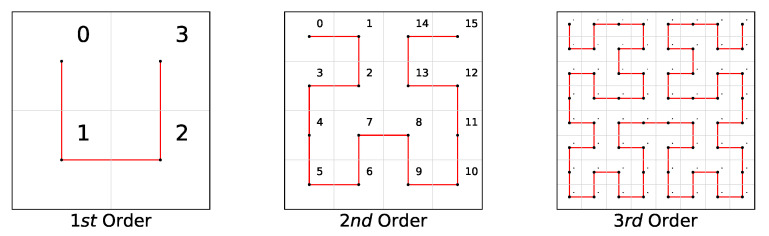
Hilbert curve.

As shown in Figure 1, the Hilbert curve is a space-filling curve. Based on the properties of these curves, each discrete cell can be traversed exactly once in a linear sequence. Furthermore, each cell can be assigned a linear order and encoded, with the encoding serving as a unique identifier for that cell. Since the Hilbert encoding avoids large discontinuities, the aggregation performance of the Hilbert space arrangement is better, i.e., points adjacent on the Hilbert curve correspond to adjacent points in the original space in the physical world. The process of generating Hilbert curves generates each new order The generation of Hilbert curves proceeds by recursively decomposing the region and applying rotation and reflection transformations to generate each new order. At each recursion, the current region is divided into four subregions, each undergoing specific transformations. The specific steps for generating a Hilbert Curve are as follows.

1. Recursive partitioning: the current region is divided into four subregions, forming an arrangement similar to the letter “E”.
2. Transformation of the subregions:
  - Apply a 90° counterclockwise rotation to the first subregion (lower left).
  - The second subregion (upper left) remains unchanged.
  - The third subregion (upper right part) remains unchanged.
  - Apply a clockwise rotation of 90° to the fourth subregion (lower right part).
3. By constant recursion, higher-order Hilbert curves are generated, and these transformations refine the curves.

### 3.2. Distributed Point Functions

**Figure 2:**
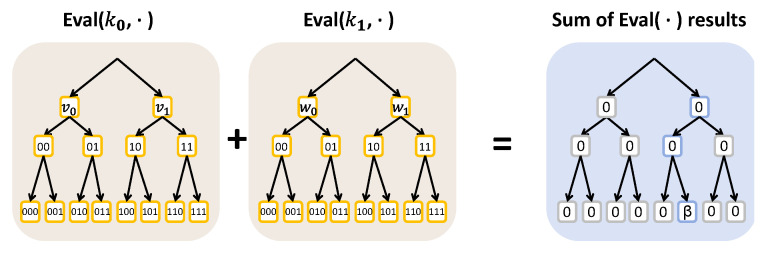
DPF with depth n=3 and designated point α=101 using the value β.

As a particular instance of function secret sharing, the distributed point functions (DPFs) [24], denoted by $f_{\alpha ,\beta }$, are defined such that the value β is non-zero exclusively when the input equals a specific point α and yields a value of zero for any other inputs. An illustrative example with depth 3 is presented in Figure 2. More precisely, in a standard two-party DPF construction defined over a finite field F, two primary algorithms are included, detailed as follows.

- Gen ($1^{\lambda },\alpha ,\beta$) → ($k_{0},k_{1}$). Given the security parameter λ, an index $\alpha \in {\left\{0,1\right\}}^{n}$, and a value β∈F, the algorithm produces a pair of DPF keys. Conceptually, the two keys jointly encode a $2^{n}$-dimensional vector over β∈F whose α-th entry equals β while all other entries are zero.
- Eval ($i,k_{i},x$) →F. Given i∈{0,1} as the party identifier, $k_{i}$ as the corresponding key and $x\in {\left\{0,1\right\}}^{n}$ as an input, the algorithm outputs the *i*-th share of the vector entry at position *x*. The correctness property of a two-party DPF, given ($k_{0},k_{1}$) ←*Gen*($1^{\lambda },\alpha ,\beta$), is formalized as
  $$\mathrm{Eval}\;\left(0,k_{0},x\right)\;+\;\mathrm{Eval}\;\left(1,k_{1},x\right)=\left\{\begin{array}{cc} \beta & \mathrm{if}\;x=\alpha \\ 0 & \mathrm{otherwise} \end{array}\right.$$

It is important to emphasize that the addition operation is carried out over the finite field F. Informally, the security guarantee of the DPF ensures that an adversary possessing only one of the keys, $k_{0}$ or $k_{1}$, gains no knowledge regarding the point α or its corresponding value β.

## 4. Models and Goals

This section introduces the system model and the threat model, followed by the design goals and an overview of the workflow.

### 4.1. Models

**Figure 3:**
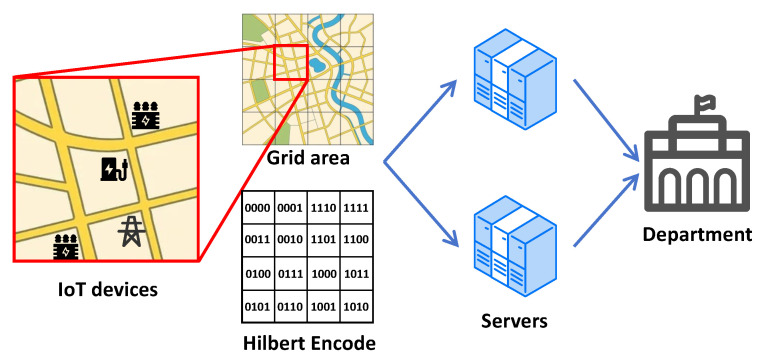
System model.

**System Model.** As shown in Figure 3, illustrated with the second-order Hilbert curve as an example, the system model comprises three main types of entities: IoT devices, two collaborative servers, and the electricity management department.

- IoT devices: The first entity consists of IoT devices widely distributed across the power grid. These devices integrate sensing and communication modules, enabling the continuous acquisition of local electrical data such as voltage, current, and load fluctuations. The collected data is pre-processed locally on the IoT device and encrypted using DPF based on the Hilbert curve encoding. The Hilbert code is mapped from the real geographic location of IoT devices. The encrypted data is then transmitted to the collaborative servers for further analysis. This allows accurate identification of areas where distribution anomalies or imbalances may exist.
- Two Collaborative Servers: The two servers work together to process the encoded data received from the IoT devices. These servers handle data processing and ensure secure transmission of results to the electricity management department. They guarantee that data is processed and transmitted securely, preserving its integrity throughout the entire procedure.
- Electricity Management Department: This entity is responsible for overseeing the operation and stability of the power grid. Once the servers provide the processed data on power distribution across the grid, the electricity management department uses this information to monitor grid performance and optimize resource allocation to ensure effective grid management. The department’s main objective is to ensure the uninterrupted and efficient functioning of the grid while maintaining stable energy distribution.

Note that this paper does not focus on detecting specific load imbalances or electrical faults with a high granularity, but rather aims to analyze broader power distribution patterns across the grid. This broader analysis serves as a critical first step to ensure efficient power distribution and early detection of potential problems, enabling better resource allocation and faster response.

### 4.2. Workflow

The main problem addressed by our scheme is power distribution analysis in large-scale power grids, specifically the identification of regions with potential power distribution issues. Traditional methods can struggle when applied to large or dynamic grids, especially when dealing with fluctuating energy demands or decentralized generation sources. Rather than pinpointing specific faults, our goal is to analyze broader regions of interest within the grid to enable timely interventions and optimize energy flow.

The general workflow of the proposed scheme is outlined as follows.

- Data Collection and Encoding: IoT devices—such as electric vehicle charging stations, smart meters, and distribution transformer monitors—are deployed across different regions of the grid to collect electrical data. These devices continuously monitor local conditions, capturing parameters like voltage and current. To enable efficient spatial analysis, the geographical area is first partitioned into smaller, manageable regions. The data from each region is then encoded using a Hilbert curve, a technique that effectively captures spatial relationships within the grid. This encoding method facilitates more accurate and scalable analysis of distribution patterns.
- Secure Transmission: Once the data is encoded, the IoT devices use DPF to generate a shared secret key for secure data transmission. The DPF ensures that the information related to fault-prone areas is encrypted and protected during transmission. The encoded data, along with the corresponding DPF shares, is securely transmitted to the two collaborative servers.
- Data Processing: The two collaborative servers receive the encrypted data and the shared keys. They work together to process the information and identify fault-prone regions based on the data provided. The servers perform the necessary computations while adhering to the security and privacy requirements set by the DPF mechanism.
- Action by the Electricity Management Department: The department analyzes the data to identify areas of the grid requiring optimization or adjustment, such as regions exhibiting imbalances or inefficiencies. After identifying such regions, the electricity management department takes the necessary actions to optimize the power distribution. This could include adjusting load distribution, deploying resources, or making other decisions to ensure the grid remains stable and efficient.

Through this approach, our scheme seeks to offer a dependable and efficient method for analyzing power distribution in modern grids, facilitating prompt responses to distribution challenges and enhancing overall grid management.

## 5. Methodology

**Figure 4:**
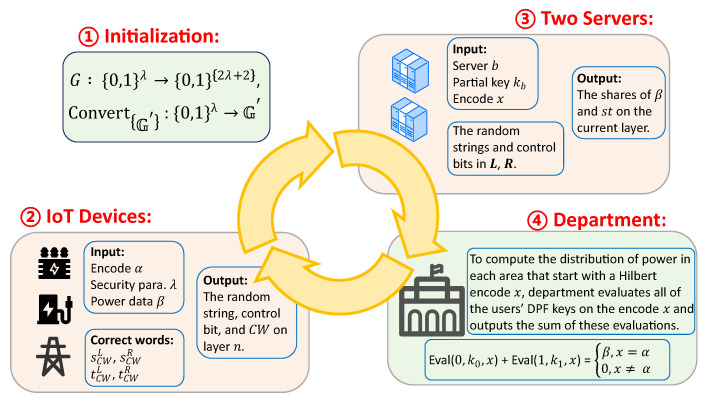
The workflow of PSDA.

This section outlines the structural design of the proposed scheme, including encoding, key generation, and data processing. We assume that the IoT devices have already collected data from the smart grid. The workflow is shown in Figure 4.

### 5.1. Encoding

In the process of encoding the Hilbert curve, the goal of each IoT device is to map each point on a given grid of size n×n to a unique binary representation, determining the corresponding code for its physical location. This encoding method is particularly suitable for efficiently partitioning and representing spatial data, especially in environments with a large number of distributed IoT devices. Initially, each IoT device obtains the grid size *n*, which is typically a power of 2, allowing the grid to be recursively divided into smaller areas.

To begin the encoding, an initial scale n/2 is set, where *n* is the grid size. At this scale, the grid is divided into four smaller quadrants. For each point (x,y) in the grid (which may represent a location containing multiple IoT devices), each device calculates a unique index by recursively examining the bit values of *x* and *y* at each scale level. This process involves the use of the bit extraction function, bit(x,scale), which extracts the relevant bit from the binary representation of the coordinate at the current scale. Specifically, bit(x,scale) is defined as

$$\mathrm{bit}\left(x,scale\right)=\left\lfloor \frac{scale}{x}\right\rfloor \;\mathrm{mod}\;2$$

To ensure that the transformation does not alter the original coordinates, temporary variables $x_{temp}$ and $y_{temp}$ are introduced. These variables hold the current values of *x* and *y* for each level of the recursive division. The values of $x_{temp}$ and $y_{temp}$ are updated at each recursive step to reflect the transformations that occur, while the original coordinates *x* and *y* remain unchanged for further computations.

Subsequently, the index is updated by the following formula:

index←index+scale×scale((3×rx)⊕ry),

where ⊕ denotes the XOR operation, which helps determine the point’s position on the Hilbert curve based on the relative values of *x* and *y* at each scale. When ry=0, the IoT devices swap *x* and *y*; when rx=1, the coordinates are reversed (i.e., x,y←(n−1−x),(n−1−y)). These transformations ensure the correct direction of the curve.

This recursive process halves the scale at each step until reaching the minimum scale (i.e., scale 1), at which point the coordinates are fully mapped onto the Hilbert curve. Ultimately, the IoT device outputs a binary code for its own point (x,y) that represents the point’s position on the curve. In this way, the Hilbert curve encoding allows IoT devices to efficiently divide the grid into smaller regions, accurately determine their location, and assign a unique identifier to each point. For details, refer to Algorithm 1.
**Algorithm 1** Generating Binary Encoding for the n×n Hilbert Curve1:**Input:** Area size n×n (assumed $n=2^{m}$), Point coordinates $\left(x,y\right)\in {\left\{0,...,n-1\right\}}^{2}$2:**Output:** Binary encoding of each point on the Hilbert curve3:**For each point **(x,y)** do**4:index←05:scale←n/26:$x_{temp}\leftarrow x$, $y_{temp}\leftarrow y$7:**while **scale>0** do**8:      $rx\leftarrow \mathrm{bit}(x_{temp},scale)$9:      $ry\leftarrow \mathrm{bit}(y_{temp},scale)$10:    index←index+scale×scale((3×rx)⊕ry)11:    **if** ry=0 **then**12:        **if** rx=1 **then**13:             $\left(x_{temp},y_{temp}\right)\leftarrow \left(n-1-x_{temp},n-1-y_{temp}\right)$14:        **end if**15:        $\left(x_{temp},y_{temp}\right)\leftarrow \left(y_{temp},x_{temp}\right)$16:    **end if**17:    scale←scale/218:**end while**19:Output binary representation of index

### 5.2. Key Generation

First, Algorithm 2 defines a pseudo-random generator *G* : ${\left\{0,1\right\}}^{\lambda }\to {\left\{0,1\right\}}^{2\lambda +2}$ and a random group element in G that converts a random string via ${\mathrm{Convert}}_{G^{\prime }}$ : ${\left\{0,1\right\}}^{\lambda }\to G^{\prime }$, where $G^{\prime }={\left\{0,1\right\}}^{\lambda }\times G$, and the random string is of length λ.
**Algorithm 2** Init1:Let $G:{\left\{0,1\right\}}^{\lambda }\to {\left\{0,1\right\}}^{2\lambda +2}$ a random generator.2:Let $G^{\prime }={\left\{0,1\right\}}^{\lambda }$, where${\left\{0,1\right\}}^{\lambda }$ employs bitwise addition3:and ${\mathrm{Convert}}_{G^{\prime }}:{\left\{0,1\right\}}^{\lambda }\to G^{\prime }$ denote a function that transforms a λ-bit random string to a random group element in G. ${\mathrm{Convert}}_{G}\left(m\right)$:4:Let u←|G|5:**if **$u=2^{s}$ for an integer *s* **then**6:     Output the group element corresponding to G(m), for PRG $G:{\left\{0,1\right\}}^{\lambda }\to {\left\{0,1\right\}}^{s}$7:**else**8:     Let $l\leftarrow \lceil \log_{2}u\rceil +\lambda$9:     Output the group element corresponding to G(m)modu, for a PRG $G:{\left\{0,1\right\}}^{\lambda }\to {\left\{0,1\right\}}^{l}$10:**end if**

In the key generation process, each IoT device is responsible for generating a pair of cryptographic keys to secure subsequent data transmission. Given the inputs λ (security parameter), α (a binary sequence of length $l=2log_{2}\left(n\right))$, β (an additional parameter for key generation), and G, the IoT device first initializes the binary sequence and two randomly generated bit strings $s_{0}^{\left(0\right)}$ and $s_{1}^{\left(0\right)}$, each of length λ. Additionally, it initializes $t_{0}^{\left(0\right)}$ and $t_{0}^{\left(1\right)}$, where $t_{0}^{\left(1\right)}$ is set to be the complement of $t_{0}^{\left(0\right)}$.

The IoT device then iteratively processes the key material through *l* iterations, applying a recursive construction. In each iteration *i*, it expands the previous state $s_{0}^{\left(i-1\right)}$ and $s_{1}^{\left(i-1\right)}$ using the pseudo-random generator *G*, resulting in four new components per key: $s_{0}^{L}$,$t_{0}^{L}$,$s_{0}^{R}$,$t_{0}^{R}$ for the first key and $s_{1}^{L}$,$t_{1}^{L}$,$s_{1}^{R}$,$t_{1}^{R}$ for the second key. Based on the current bit $\alpha_{i}$, it determines whether to retain the left *L* or right *R* portion, assigning them to Keep and Lose variables accordingly. The lost portion is used to generate a correlation term $s_{CW}$ as the XOR of the discarded values from both keys. Similarly, $t_{CW}^{L}$ and $t_{CW}^{R}$ are computed based on the previous values, incorporating $\alpha_{i}$ and a constant offset to ensure correct correlation. These are then concatenated to form $CW^{i}$, representing that iteration’s correlated component.

After computing $CW^{\left(i\right)}$, each IoT device updates $s_{b}^{\left(i\right)}$ and $t_{b}^{\left(i\right)}$ for both keys, modifying them based on their previously retained values and their relationship with the newly computed correlation terms. This iterative process continues until all *l* bits of α have been processed. In the final step, the device computes $CW^{l+1}$ using the last state value $t_{1}^{\left(l\right)}$, adjusting it with β and the conversion outputs of the last key states. Finally, the full key pair $\left(k_{0},k_{1}\right)$ is assembled, consisting of the initial seeds and all the correlation terms computed during the iterations. For details, refer to Algorithm 3.

### 5.3. Data Processing

In this step, the servers evaluate the key material and determine the fault-prone areas based on the received encoded data. Algorithm 4 gives the detailed steps. The servers first construct the key $k_{b}$, which includes the initial state values $s^{\left(0\right)}$ and $t^{\left(0\right)}$, and the correlation values $CW^{\left(1\right)},CW^{\left(2\right)},\ldots ,CW^{\left(l+1\right)}$. This key is essential for the servers to securely process the data and determine the relevant fault-prone regions.

For each iteration *i* ranging from 1 to *l*, the servers compute the correlation term $CW^{\left(i\right)}$ using the previous key values $s_{CW}$, $t_{CW}^{L}$, $t_{CW}^{R}$, which represent the left and right components of the correlation data. The servers then calculate $\tau_{i}$, which is derived from the pseudo-random generator $G(s^{\left(i-1\right)})$, adjusted by the product of $t^{\left(i-1\right)}$ and the concatenated correlation values [$s_{CW}^{L}$||$t_{CW}^{L}$||$s_{CW}^{R}$||$t_{CW}^{R}$]. This result is a combination of the new state values $s^{L},t^{L},s^{R},t^{R}$, each of length 2λ+2, which are used for further processing.
**Algorithm 3** Gen (λ,α,β,G)1:$\alpha =\alpha_{1},\ldots ,\alpha_{\ell }\in {\left\{0,1\right\}}^{l}$2:$s_{0}^{\left(0\right)}\leftarrow {\left\{0,1\right\}}^{\lambda }$, $s_{1}^{\left(0\right)}\leftarrow {\left\{0,1\right\}}^{\lambda }$3:$t_{0}^{\left(0\right)}\leftarrow {\left\{0,1\right\}}^{\lambda }$, $t_{0}^{\left(1\right)}\leftarrow 1^{\lambda }⊕t_{0}^{\left(0\right)}$4:**for **i=1 to *l* **do**5:      $s_{0}^{L}\left|\right|t_{0}^{L}\left|\right|s_{0}^{R}\left|\right|t_{0}^{R}\leftarrow G\left(s_{0}^{\left(i-1\right)}\right)$, $s_{1}^{L}\left|\right|t_{1}^{L}\left|\right|s_{1}^{R}\left|\right|t_{1}^{R}\leftarrow G\left(s_{1}^{\left(i-1\right)}\right)$6:      **if** $\alpha_{i}=0$ **then**7:           Keep ←L, Lose ←R8:      **else**9:           Keep ←R, Lose ←L10:    **end if**11:    $s_{CW}\leftarrow s_{0}^{\mathrm{Lose}}⊕s_{1}^{\mathrm{Lose}}$12:    $t_{CW}^{L}\leftarrow t_{0}^{L}⊕t_{1}^{L}⊕\alpha_{i}⊕1^{\lambda },t_{CW}^{R}\leftarrow t_{0}^{R}⊕t_{1}^{R}⊕\alpha_{i}$13:    $CW^{\left(i\right)}\leftarrow s_{CW}\left|\right|t_{CW}^{L}\left|\right|t_{CW}^{R}$14:    $s_{b}^{\left(i\right)}\leftarrow s_{b}^{\mathrm{Keep}}⊕t_{b}^{\left(i-1\right)}\cdot s_{CW}$, for b=0,115:    $t_{b}^{\left(i\right)}\leftarrow t_{b}^{\mathrm{Keep}}⊕t_{b}^{\left(i-1\right)}\cdot t_{CW}^{Keep}$, for b=0,116:**end for**17:$CW^{\left(l+1\right)}\leftarrow {\left(-1\right)}^{t_{1}^{l}}\left[\beta -\mathrm{Convert}\left(s_{0}^{l}\right)+\mathrm{Convert}\left(s_{1}^{l}\right)\right]$18:Let $k_{b}\leftarrow s_{b}^{\left(0\right)}\left|\right|t_{b}^{\left(0\right)}||CW^{\left(1\right)}||\ldots ||CW^{\left(l+1\right)}$19:**return **$\left(k_{0},k_{1}\right)$

**Algorithm 4** Eval (*b*, $k_{b}$, *x*)


$k_{b}=s_{b}^{\left(0\right)}\left|\right|t_{b}^{\left(0\right)}||CW^{\left(1\right)}||\ldots ||CW^{\left(l+1\right)}$

**for **i=1 to *l* **do**      $CW^{\left(i\right)}\leftarrow s_{CW}\left|\right|t_{CW}^{L}\left|\right|t_{CW}^{R}$      $\tau_{i}\leftarrow G\left(s_{b}^{\left(i-1\right)}\right)⊕(t_{b}^{\left(i-1\right)}\cdot [s_{CW}^{L}\left|\right|t_{CW}^{L}\left|\right|s_{CW}^{R}\left|\right|t_{CW}^{R}])$      $\tau_{i}=s_{b}^{L}\left|\right|t_{b}^{L}\left|\right|s_{b}^{R}\left|\right|t_{b}^{R}\in {\left\{0,1\right\}}^{2\lambda +2}$      **if** $x_{i}=0$ **then**            $s_{b}^{\left(i\right)}\leftarrow s_{b}^{L}$, $t_{b}^{\left(i\right)}\leftarrow t_{b}^{L}$      **else**            $s_{b}^{\left(i\right)}\leftarrow s_{b}^{R}$, $t_{b}^{\left(i\right)}\leftarrow t_{b}^{R}$      **end if**
**end for**


$y_{b}\leftarrow {\left(-1\right)}^{b}\left[\mathrm{Convert}\left(s_{b}^{\left(l\right)}\right)+t_{b}^{\left(l\right)}\cdot CW^{\left(l+1\right)}\right]$


**return** $y_{b}$




Depending on the value of the binary input $x_{i}$, which indicates whether to select the left or right portion of the computed values, the servers update the current state values. If $x_{i}=0$, the servers set $s^{\left(i\right)}$ and $t^{\left(i\right)}$ to $s^{L}$ and $t^{L}$, respectively. If $x_{i}=1$, they select $s^{R}$ and $t^{R}$ as the new state values. This selection process ensures that the correct path is followed during the key evaluation, depending on the fault localization result.

Upon completing all iterations, the servers compute the final output $y_{b}$, which is determined by the binary value *b*. The final output is derived by combining the final states $s^{\left(l\right)}$ and $t^{\left(l\right)}$ with the correlation value $CW^{\left(l+1\right)}$. The conversion of $s^{\left(l\right)}$ and $t^{\left(l\right)}$ along with the multiplication of $t^{\left(l\right)}$ and $CW^{\left(l+1\right)}$ determines the final output that is used to identify the fault-prone region. The servers then return the output $y_{b}$, which is the result of the data processing and fault evaluation. This result is forwarded to the department, allowing them to take appropriate actions based on the identified areas of electricity distribution.

**Figure 5:**
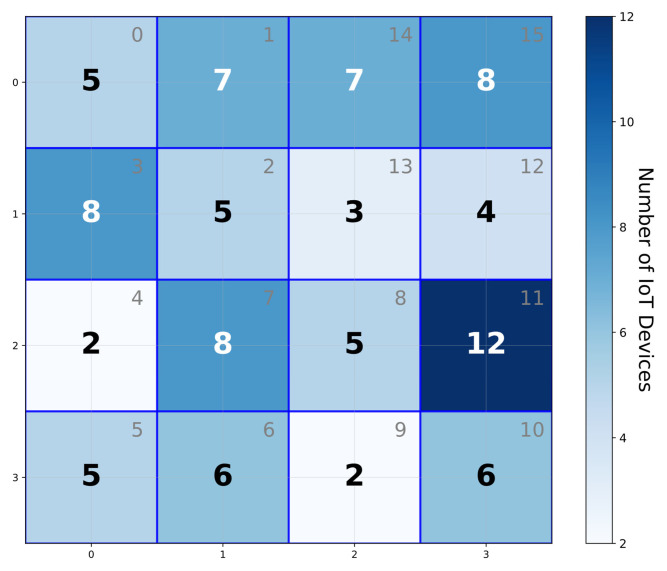
IoT device count distribution by Hilbert regions.

**Figure 6:**
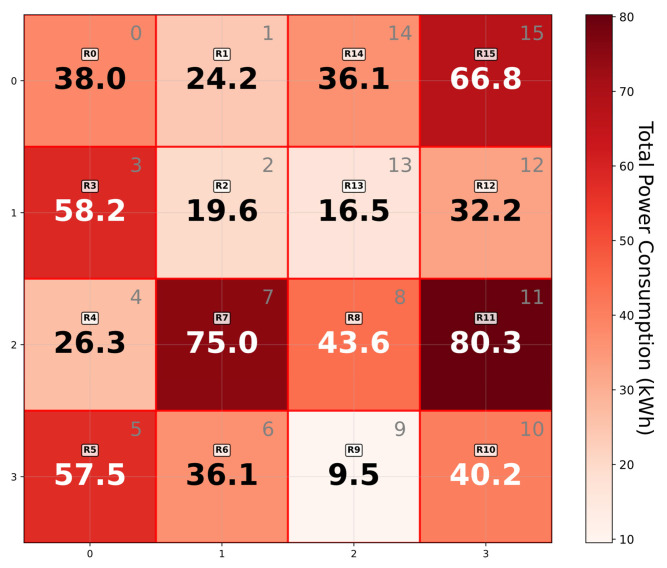
Aggregated power consumption by Hilbert regions.

Finally, the electricity management department collects the evaluation results $y_{0}^{i}$ and $y_{1}^{i}$ for each IoT device *i* from two collaborative servers. Then, the electricity management department aggregates these results across all IoT devices within each Hilbert region to obtain the region’s actual measurement values. Figure 5 and Figure 6 provide a visual representation of the aggregated results used for grid management.

### 5.4. Security Analysis

**Threat Model.** The PSDA involves three types of entities: a set of IoT devices D deployed across the smart grid, two non-colluding servers $S_{0}$ and $S_{1}$, and an electricity management department M.

- IoT devices: Each IoT device is assumed to be semi-honest. That is, the device follows the prescribed protocol to generate and transmit its encoded data correctly but may attempt to infer additional information from the messages it handles. Devices do not collude with other devices or with the servers. In practice, a subset of devices may behave maliciously. A malicious IoT device may tamper with its reported data, thereby influencing the overall power analysis results. The primary objective of our work, however, is to assist the electricity management department in promptly detecting such anomalous data and conducting further investigation and verification. Therefore, the security analysis in this paper does not consider scenarios in which IoT devices behave maliciously.
- Two non-colluding servers: The two servers $S_{0}$ and $S_{1}$ are assumed to be semi-honest. They faithfully follow the protocol execution but may attempt to learn private information from their locally stored shares. The fundamental assumption is that these servers are non-colluding, typically operated by the electricity management department or government. If collusion occurs, the DPF-based privacy guarantee no longer holds.
- Electricity management department: The management department M is regarded as an honest party that aggregates the computation results received from both servers. It is trusted to perform the aggregation correctly.

**Adversarial Capabilities.** We consider the following types of potential adversarial behaviors within the PSDA.

- Multi-device adversary: This adversary compromises an arbitrary subset of IoT devices, obtaining access to their local measurements, encryption keys, and DPF shares. The compromised devices may collude with each other and attempt to infer information about other uncompromised IoT devices or the servers.
- Single-server adversary: This adversary corrupts one of the two non-colluding servers (either $S_{0}$ or $S_{1}$), gaining full access to its stored data, computation states, and received DPF shares. The adversary may attempt to infer the private information of IoT devices or the internal data of the another server.

It is further possible that both adversarial behaviors occur simultaneously, i.e., an adversary compromises one server and multiple IoT devices at the same time. In such a case, the adversary is assumed to have access to the compromised server’s internal states as well as the plaintext data, encryption keys, and DPF shares of the compromised devices, and it may jointly analyze these to infer information about other uncompromised IoT devices or the remaining honest server.

**Theorem** **1.** *Under the threat model definition above, and letting $F_{\mathrm{PSDA}}$ denote the ideal functionality, the real protocol $\Pi_{\mathrm{PSDA}}$ securely realizes $F_{\mathrm{PSDA}}$ against any PPT adversary corrupting an arbitrary subset of devices and at most one server. This ensures that no entity can learn extra sensitive information from the data obtained.*

**Proof.**  Under the defined threat model, the PSDA framework preserves data privacy and confidentiality against all considered adversarial capabilities. Each IoT device locally encodes its power distribution data and transmits only the corresponding DPF shares to the two non-colluding servers. Server $S_{0}$ stores one share $k_{0}$, while Server $S_{1}$ retains the complementary share $k_{1}$. Due to the inherent properties of the DPF construction, neither share alone reveals any meaningful information about the underlying plaintext values, as both key components are computationally indistinguishable from random strings without the corresponding counterpart. Consequently, even if one server is fully compromised, the adversary can access only partial, non-informative data that cannot be used to reconstruct or infer the original measurements of any IoT device or the secret state of the other server.

When multiple IoT devices are compromised, the adversary may obtain the plaintext data, encryption keys, and DPF shares corresponding to those devices. However, such compromise yields no additional advantage in inferring information about other uncompromised devices, since the encoding and key generation processes are performed independently per device. The DPF outputs generated by distinct devices are uncorrelated, and therefore knowledge of one device’s data or shares does not enable inference of another’s encoded values or communication content.

Furthermore, even in the scenario where both adversarial behaviors occur simultaneously—namely, when one server and multiple IoT devices are compromised—the confidentiality of the remaining honest entities remains preserved. In this case, the adversary has full access to the internal states of the compromised server and the complete data of the corrupted devices but cannot derive any additional information about other devices or the remaining honest server. This is because the DPF encoding of each device is independently randomized, and the unavailability of the complementary key share prevents the reconstruction of any unexposed power distribution data.

Finally, the electricity management department only receives the aggregated computation results derived from both servers and is unable to access any individual-level data. Since all transmitted values are processed under the DPF framework and the aggregation phase does not involve decryption of raw data, it is computationally infeasible to extract or infer sensitive information beyond the intended aggregate statistics. Therefore, under the defined threat model, the PSDA ensures that no party including compromised servers, compromised devices, or their combination can obtain additional private information beyond what is explicitly revealed by the final aggregated output. □

## 6. Results

### 6.1. Implementation and Settings

The PSDA scheme is designed to tackle challenges related to power distribution analysis and data security in modern smart grid environments. In particular, it facilitates the identification of regions prone to power distribution issues within IoT-enabled smart grids, while safeguarding sensitive data through encryption mechanisms and DPF. To assess the performance and efficiency of the proposed method, we conducted experiments assessing the computational and communication overheads associated with each stage of the scheme, including data uploading, processing, and aggregation. These experiments enable a thorough comparison of PSDA’s performance against conventional power distribution analysis methods, offering insights into its practical applicability for large-scale deployments. All experiments were conducted on a laptop featuring a 13th Gen Intel (R) Core (TM) i9-13900HX CPU (2.20 GHz), NVIDIA GeForce RTX 4070 GPU with 8 GB VRAM and 16 GB of RAM, running 64-bit Windows 11, with Python 3.13 (Manufacturer: HP Inc.; City: Beijing; Country: China). Additionally, to provide a consistent privacy baseline, all methods are evaluated under equivalent security parameters. In our scheme, the use of distributed point functions (DPFs) guarantees privacy protection comparable to standard cryptographic primitives, and all experiments assume a security level equivalent to 128-bit security. This setup ensures that the reported computational and communication overheads are directly comparable while maintaining equivalent privacy guarantees across all methods.

### 6.2. Metrics and Baselines

PSDA’s performance is evaluated based on its computational cost, communication overhead, and aggregation time. The metrics employed for this comparison are defined as follows.

- Shruti_FEISG [20]: This paper proposed an encryption-based data consolidation strategy for smart grids leveraging fog computing [25,26], which shifts part of the cloud’s computation and storage tasks to fog nodes located near smart meters. By enabling local data compression and consolidation, the approach reduces transmission costs and improves efficiency while maintaining data security. Data is first processed and compressed at smart meters, and then it is aggregated at fog devices before being selectively uploaded to the cloud. Though this protects privacy and uses fog nodes to reduce the cost of communication with the server, it incurs significant computational cost.
- Rostampour_EPSG [21]: This paper proposed a lightweight authentication scheme for smart grids that leverages the unique physical characteristics of devices to ensure the integrity and authenticity of smart meters. By preventing cloning and tampering at the device level, the scheme establishes a secure foundation for grid communication. It provides strong protection against common attacks. While it offers robust privacy protection, it does incur certain computational and communication costs.
- Feng_Panther [27]: This paper proposed Panther, a practical secure two-party neural network (2P-NN) inference system that enables clients to obtain inference results from a server-hosted deep neural network without revealing their inputs, while the server’s model parameters remain private. The system combines a customized homomorphic encryption scheme for efficient linear-layer computation with an optimized millionaires’ protocol based on oblivious transfer and secret sharing for nonlinear functions such as ReLU and max-pooling. By reducing polynomial multiplications and communication rounds, Panther significantly decreases both computation and communication overhead. While it achieves state-of-the-art efficiency compared with prior works, it still uses homomorphic encryption as a privacy protection technique, which causes significant computational and communication overhead.
- PSDA refers to the efficient and privacy-preserving spatial distribution statistics scheme introduced in Section 5.

### 6.3. Performance Evaluation

The schemes are assessed by examining the communication and computational resources required to accomplish the same localization objective. Specifically, our experiments evaluate the performance of different schemes across three key metrics: the computation time for IoT devices to upload data, the communication overhead incurred during data upload, and the aggregation time at the power management department. Two key parameters are considered in these experiments: the number of IoT devices and the encoding length, where the encoding length corresponds to the number of spatial regions. When assessing the impact of varying IoT device numbers, the encoding length is fixed at 16. Conversely, when evaluating the effect of different encoding lengths, the number of IoT devices is fixed at 200. This design allows us to isolate and analyze the influence of each parameter on system performance.

#### 6.3.1. Computation Costs on Uploading

**Figure 7:**
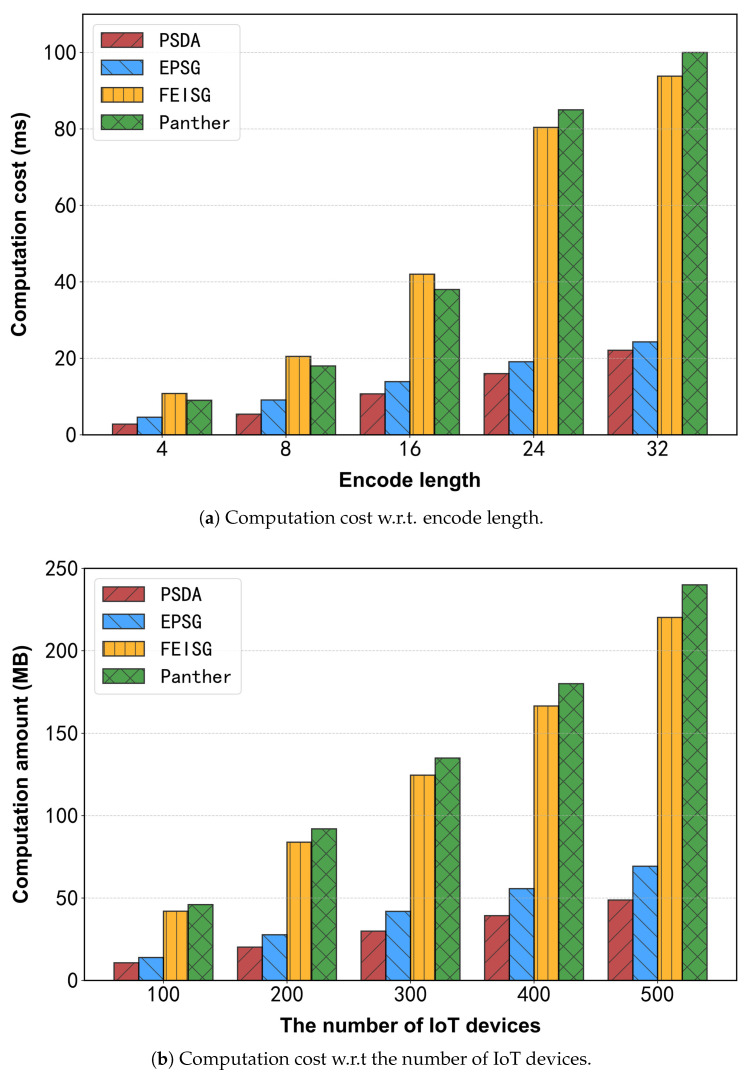
Experimental results for computation cost.

The computational overhead incurred by the IoT devices is first analyzed. As depicted in Figure 7, the comparison evaluates performance across different string lengths (corresponding to area identifiers) and varying numbers of IoT devices.

As shown in Figure 7a, all schemes’ uploading time increases approximately linearly with the encoder length. This trend arises because longer encoders require additional processing, resulting in higher computational overhead. PSDA demonstrates the highest efficiency due to its use of DPF. In contrast, both Shruti_FEISG and Rostampour_EPSG must encrypt each region code prior to uploading, substantially increasing their time overhead. From Figure 7b, it is evident that the uploading time for all schemes exhibits a linear increase in response to the increasing number of IoT devices, which stems from the larger volume of data requiring processing. Notably, the proposed PSDA consistently achieves superior computational efficiency compared to alternative approaches. The underlying reason is that Shruti_FEISG and Rostampour_EPSG generate their keys via linear encryption, leading to relatively low efficiency in key generation. In both scenarios, Feng_Panther generated a significant amount of computation due to homomorphic encryption. In contrast, PSDA incorporates an optimized computation strategy, thereby reducing overall overhead.

#### 6.3.2. Communication Costs upon Uploading

**Figure 8:**
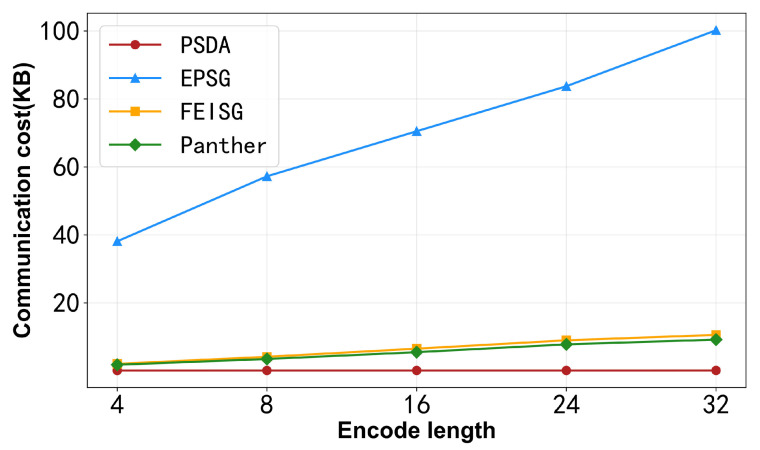
Communication cost w.r.t. encode length.

We then assess the communication costs incurred by the remote sensing entity, with Figure 8 illustrating the comparison for varying string lengths.

From the experimental results, it can be seen that as the encode length increases, the communication costs of all schemes show a linear growth trend. This is because longer encode length requires more ciphertext size and operations. As can be seen in Figure 8, the communication cost of PSDA is the lowest, followed by Shruti_FEISG, and Rostampour_EPSG has the most significant costs. Shruti_FEISG uses fog nodes to effectively reduce communication overhead, but compared with PSDA, FEISG encrypts each region code and then uploads it, and the ciphertext size is very large; meanwhile, Rostampour_EPSG does not use any special methods, which undoubtedly increases the communication overhead in the smart grid architecture based on IoT devices.

#### 6.3.3. Aggregation Time

**Figure 9:**
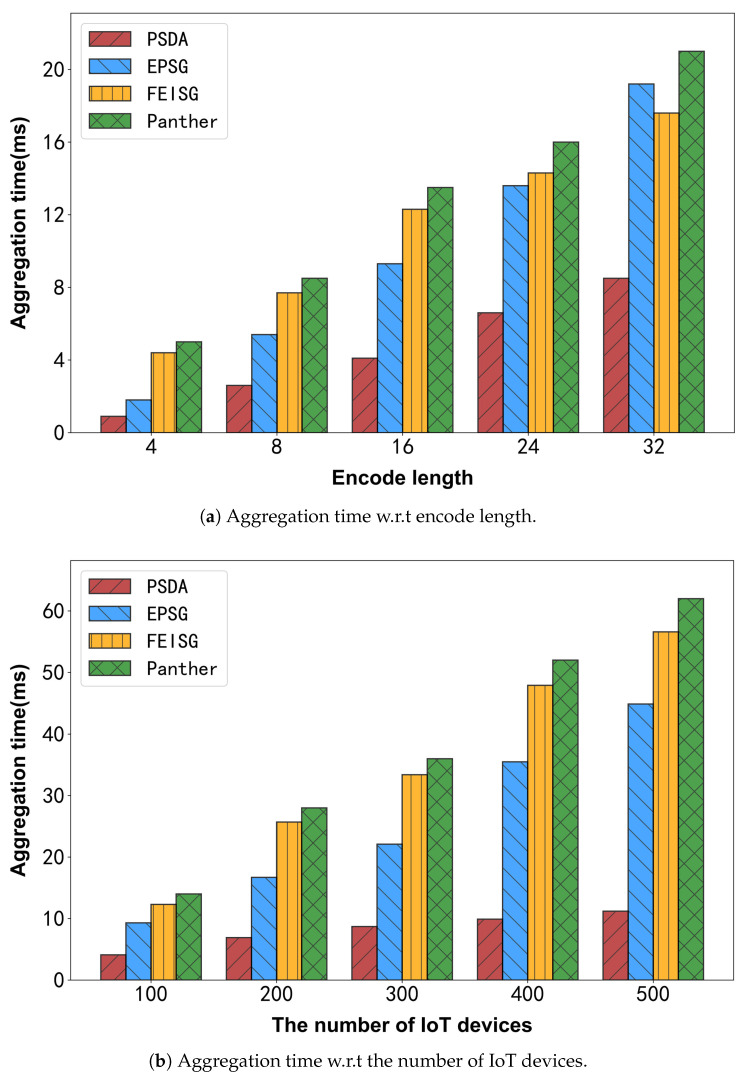
Experimental results for aggregation time.

Finally, the aggregation time on the department side is evaluated. Figure 9 illustrates the comparison results using different numbers of IoT devices and varying encoding lengths.

As illustrated in Figure 9a, the aggregation time of all schemes increases roughly linearly with the encoder length. This is because longer code lengths require more regions to be processed, and more data needs to be processed when performing power distribution analysis, resulting in higher time overhead. PSDA is the most efficient because it uses DPF. In the final data aggregation stage, only the data sent by the server needs to be added. In addition, since both Shruti_FEISG and Rostampour_EPSG need to decrypt the ciphertext corresponding to each area code before aggregation, these operations greatly increase the time overhead. As illustrated in Figure 9b, the aggregation time of all schemes increases approximately linearly with the growth in the number of IoT devices, primarily due to the larger volume of requests requiring processing. It is worth emphasizing that the proposed PSDA consistently surpasses other schemes in computational efficiency. This advantage arises because Shruti_FEISG and Rostampour_EPSG employ linear encryption for key generation and require linear-time decryption, and Feng_Panther uses homomorphic encryption to reach its security goal, which leads to the lowest aggregation efficiency. In contrast, PSDA is specifically designed to optimize the aggregation phase, thereby minimizing time overhead.

#### 6.3.4. Experimental Summary

The experimental results indicate that PSDA consistently surpasses other schemes in terms of computational efficiency, communication overhead, and aggregation time. As the encoding length and number of IoT devices increase, PSDA maintains the lowest overhead and communication costs. In comparison, Shruti_FEISG, Rostampour_EPSG, and Feng_Panther incur higher computational and communication overhead. PSDA provides a more efficient solution for power distribution analysis in IoT-based smart grids.

## 7. Discussion

Power distribution is an unavoidable topic within the field of smart grids. In this section, we first discuss the scalability of our proposed scheme in dynamic and large-scale IoT-based smart grid environments and then present a comprehensive overview of related research on data processing and advances in function secret sharing.

### 7.1. Scalability in Dynamic and Large-Scale Smart Grids

A critical requirement for smart grid analytics is the ability to scale efficiently to millions of IoT devices and to remain robust under high-frequency data streams. Our proposed scheme addresses this challenge through the integration of Hilbert curve encoding with DPF.

Hilbert curve encoding preserves spatial locality, which means that when IoT devices are mobile or dynamically reallocated across the network, only localized updates to their spatial representation are required. This property enables rapid re-encoding and seamless adaptation of the system without incurring significant computational or communication overhead. Such efficiency is particularly advantageous in scenarios where IoT devices frequently change their physical locations, such as mobile sensors or vehicular energy systems.

Meanwhile, DPF ensures privacy-preserving aggregation with lightweight cryptographic operations. Its communication complexity grows only minimally with the number of devices, making it suitable for massive-scale deployments where millions of devices generate frequent measurement reports. This balance between security and efficiency allows the system to handle high-frequency real-time data streams without overwhelming the underlying communication infrastructure.

By combining Hilbert curve encoding and DPF, our scheme demonstrates strong scalability both in terms of supporting mobile devices and in coping with very large IoT deployments. This complements our experimental evaluation by clarifying the applicability of the system under demanding real-world scenarios.

### 7.2. Detection and Localization in Smart Grid

In short circuit situations, fault location can be determined using the measured current and known network impedance, known as the impedance-based method [28]. However, in earth faults, the fault impedance is variable and often unknown, complicating the method. While impedance-based methods are straightforward, they require detailed knowledge of the network [29]. A common issue is the identification of multiple potential fault locations [30]. Another method, the traveling wave technique, is highly accurate but struggles in large networks due to impedance discontinuities and reflections. It also requires specialized equipment, which may not be available during faults. IoT devices have proven useful in impedance-based methods. In [31], wavelet transform was applied to current and voltage data from distribution networks, allowing fault detection with edge computing to reduce data transmission. This local analysis minimizes data load, as high-frequency real-time measurements may overwhelm LPWAN technologies. In [32], a cloud-based IoT solution for fault detection and localization uses current sensing devices with edge devices to reduce transmitted data. The system divides the network into zones, detecting individual and simultaneous faults, with localization accuracy depending on the number of zones. Traditional fault location methods have been enhanced with AI techniques [29], though these often require large datasets for training, making them impractical in some cases. A hybrid approach integrating Discrete Wavelet Transform (DWT) with neural networks, including ANN, MLP, and ELM, for fault detection and localization is presented in [33]. Fault detection can also focus on specific devices, as seen in [34], where deep learning is applied to multi-spectral images for power IoT device fault detection. In [35], fuzzy logic and ANFIS are used for fault classification and localization in smart grids, achieving 99.9% accuracy. Additionally, in [36], the Decision Tree and Random Forest algorithms are used for fault detection, with ANFIS for localization. In [37], LSTM networks integrated with FFNNs are employed for online fault detection and localization. Additionally, ref. [38] applies machine learning techniques, including Wavelet Transform and GoogLeNet for fault classification and CNNs for localization, achieving both high accuracy and rapid processing.

### 7.3. Function Secret Sharing Applications

Function secret sharing (FSS), introduced by Elette Boyle et al. [39], is a cryptographic primitive designed to enhance the efficiency of Private Information Retrieval (PIR). It implements a novel approach by protecting function information during computation via secret sharing. Over time, FSS has been applied to Secure Multi-Party Computation (MPC) [40], with research focusing on reducing communication overhead during the online evaluation of complex nonlinear functions. This is achieved by precomputing correlated randomness in an offline phase, effectively shifting much of the communication cost from online execution to offline and local computations, thereby significantly expanding the optimization potential for secure multi-party computations.

Further developments have expanded the capabilities of FSS. For instance, Boyle et al. [24] introduced an enhanced FSS construction that supports arbitrary families of linear and nonlinear functions, ensuring security even in the presence of malicious actors while requiring only constant communication and computational effort. These improvements have significantly enhanced both the flexibility and efficiency of FSS applications.

Moreover, threshold function secret sharing (TFSS) [41], a specialized variant of FSS, has been investigated. TFSS permits only a designated subset of participants to engage in the reconstruction process, thereby offering improved adaptability and robustness in practical deployment scenarios.

## 8. Conclusions

In this paper, we propose a power distribution analysis method (PSDA) based on IoT devices and Hilbert curve encoding, which aims to analyze power distribution patterns quickly and accurately while ensuring data privacy through the use of distributed point functions (DPFs). In solving the power distribution analysis problem, the method effectively identifies regions with potential power distribution imbalances by leveraging electricity consumption data from IoT devices and efficiently aggregates the distributed IoT devices using Hilbert curves to improve data processing and transmission speed. Experimental results demonstrate that the PSDA method excels in identifying power distribution patterns with high accuracy and responsiveness, enabling rapid identification of areas with potential distribution issues. Our method provides a paradigm, and future research can further explore the application of the PSDA method in real-world grid environments and optimize system performance by integrating it with other advanced smart grid technologies, thus enhancing both operational efficiency and grid management capabilities.

## Data Availability

Data is contained within the article.
